# Outcomes of A2/A2B to B Deceased Donor Kidney Transplantation: A Retrospective Study

**DOI:** 10.7759/cureus.73368

**Published:** 2024-11-10

**Authors:** Mia A Jose, Ketan Tamirisa, Srichandra Pallerla, Debra Meeks, Anna Curtis, Kathryn Lozano, Jessica Morton, Machaiah Madhrira, Ashraf I Reyad, Sridhar R Allam

**Affiliations:** 1 Carroll Medical Academy, Carroll Senior High School, Southlake, USA; 2 Public Health, Washington University in St. Louis, St. Louis, USA; 3 Science, Technology, Engineering and Mathematics (STEM) Pathway, Heritage High School, Frisco Independent School District, Frisco, USA; 4 Nephrology, Medical City Fort Worth, Transplant Institute, Fort Worth, USA; 5 Transplant Nephrology, PPG Health, Fort Worth, USA; 6 Transplant Surgery, Medical City Fort Worth, Transplant Institute, Fort Worth, USA; 7 Internal Medicine, Burnett School of Medicine at TCU (Texas Christian University), Fort Worth, USA

**Keywords:** a2/a2b to b kidney transplantation, blood group b candidates, clinical success kidney transplants, minority candidates, post-transplant outcomes, transplant access

## Abstract

Background

A2/A2B to B kidney transplantation has the potential to increase transplant access for traditionally disadvantaged blood group B minority candidates. Despite prior reports of positive post-transplant safety and clinical success, A2/A2B to B kidney transplantation remains underutilized in the United States. This study aims to investigate the post-transplant outcomes of A2/A2B to B kidney transplants performed at our center.

Methods

A retrospective study of all A2/A2B to B deceased donor kidney transplants (DDKTs) at our center from 2017 through 2023 was performed. Recipient and donor demographics, recipient medical history, time to transplant from listing, and post-transplant clinical outcomes were assessed, including one-year graft and patient survival.

Results

Of the 54 A2/A2B to B DDKTs performed during this period, 36 recipients were male, and 18 were female. The mean recipient age was 53.2 years. There were 22 (40.7%) African American recipients, 12 (22.2%) Hispanic recipients, 11 (20.3%) Caucasian recipients, eight (14.8%) Asian recipients, and one (1.8%) recipient of “other” race. The mean estimated post-transplant survival score was 46.5%. The mean donor age was 40.2 years, and the mean kidney donor profile index score was 44%. The mean time from waitlisting to transplant was 216 days. Delayed graft function was observed in five (9.2%) patients. Three (5.5%) patients had biopsy-proven acute rejection in the first year after transplant. The mean serum creatinine at one-year post-transplant was 1.4 mg/dL. At one-year post-transplant, graft survival was 96.2%, and patient survival was 98.1%.

Conclusions

Our study demonstrated excellent one-year post-transplant graft and patient survival rates with A2/A2B to B DDKT, with minority candidates predominantly benefiting from this.

## Introduction

Blood group B candidates face disproportionately lower access to kidney transplantation compared to other blood groups [1]. Analysis of the Organ Procurement and Transplantation Network (OPTN) data reveals significant differences in wait time for kidney transplant based on blood group: 1,744 days for group B candidates, compared to 1,554 days for group O, 898 days for group A, and 463 days for group AB candidates [2]. Multiple factors may contribute to this disparity. Minority populations constitute a higher percentage of the B blood group waitlist [3]. Historically, these groups have had lower rates of living donor transplantation, making them more dependent on the deceased donor waitlist. Additionally, the pool of available donors with blood group B is smaller than other blood group types, due to lower rates of organ donation among minority groups, with blood type B being more common among them [4].

To address this problem, the kidney allocation system (KAS) in the United States (U.S.) has historically undergone two major changes. Since 2001, available blood group B kidneys were no longer allocated to blood group AB candidates. Furthermore, in 2014, A2/A2B to B deceased donor kidney transplantation (DDKT) became a part of the KAS. Under this change, A2/A2B kidneys are now preferentially allocated to B candidates. Blood group A antigen expression in kidneys from A2 donors, compared to kidneys from A1 donors, is significantly lower, rendering A2 kidneys less risky for ABO-incompatible transplantation [5]. However, not all B candidates are eligible for A2/A2B kidneys. The pioneering work of the Midwest Transplant Network (MTN) established the safety of A2/A2B to B transplants in patients with low anti-A titer, defined as less than 1:8, assessed through an easily performed blood test [2]. Despite the reported safety and feasibility, in combination with the potential to increase transplant access to traditionally disadvantaged blood group B minority candidates, A2/A2B to B DDKT remains underutilized in the U.S. [6]. This study aimed to investigate the safety and one-year post-transplant outcomes of A2/A2B to B DDKT performed at our center.

The findings of this study were previously presented as a meeting abstract at the 2024 American Transplant Congress in Philadelphia, U.S., on June 3, 2024 [7].

## Materials and methods

Our center initiated an A2/A2B to B DDKT program in 2017. All B blood group candidates had anti-A IgG titers checked twice, at least a month apart, during their kidney transplant evaluation. The methodology our hospital lab uses for testing anti-A titers was adopted from the MTN laboratory [2]. We used a test tube and non-anti-human globulin (AHG) method. Titers were done using A1 RBCs, not A2 RBCs. Rather than performing total (IgG and IgM) antibody testing, we used serum that had been treated with dithiothreitol (DTT) to estimate IgG anti-A titer. Patients with titers less than 1:8 were waitlisted as eligible for A2/A2B to B DDKT. While on the waitlist, anti-A titers were repeated every three months to remain eligible. Patient exclusion criteria included positive complement-dependent cytotoxicity (CDC) or flow cytometry human leukocyte antigen (HLA) crossmatch, anti-A titers of 1:8 or greater, patients who could not receive depleting antibody induction therapy, patients with a history of thrombotic microangiopathy, and patients with human immunodeficiency virus (HIV). Rabbit anti-thymocyte globulin was used for induction immunosuppression. A cumulative dose of 4.5 to 6 mg/kg was utilized, depending on immunological risk. Steroids were administered at 500 mg on the day of the transplant and tapered to 20 mg daily by the fourth postoperative day. All patients were maintained on a triple immunosuppressive regimen of tacrolimus (goal trough level of 5-10 ng/mL, depending on the time since transplant), mycophenolate sodium (720 mg twice daily, with the dose decreased for any adverse effects), and prednisone 5 mg daily. We kept a low threshold for transplant kidney biopsy for graft dysfunction.

A retrospective study of all recipients who received A2/A2B to B DDKT at our center from 2017 through 2023 was performed. Data collection included recipient age, gender, race, cause of end-stage renal disease (ESRD), estimated post-transplant survival (EPTS), donor age, kidney donor profile index (KDPI), time to transplant from waitlisting, delayed graft function (DGF) rates, post-transplant serum creatinine trends, results of kidney transplant biopsy performed for cause, and one-year post-transplant graft and patient survival rates. Graft failure was defined as a return to dialysis, need for retransplant, or patient death with a functioning graft. This study was approved by the Institutional Review Board (IRB) responsible for overseeing human subjects research at our institution (MCOR-01). Informed consent from patients was not obtained, as this was a retrospective study using de-identified data.

Statistical analysis

Results were reported via descriptive statistics. Statistical analyses were performed using SAS version 9.4 (SAS Institute, Cary, NC, USA). Descriptive statistics of continuous variables were presented as means or medians with ranges. Categorical variables were presented as proportions or percentages of the total sample.

## Results

Between 2017 and 2023, we performed 54 A2/A2B to B DDKTs, representing 37.5% of B blood group candidates who received kidney transplants at our center during this time. Table 1 shows the demographics of recipients and donors.

**Table 1:** Recipient and donor demographics (n = 54) SD: standard deviation; ESRD: end-stage renal disease; EPTS: estimated post-transplant survival; KDPI: kidney donor profile index

Characteristic	No. (%)
Recipient mean age, years (±SD)	53.2 (±10.8)
Recipient gender	
Male	36 (66.7%)
Female	18 (33.3%)
Recipient race	
African American	22 (40.7%)
Hispanic	12 (22.2%)
Caucasian	11 (20.3%)
Asian	8 (14.8%)
Other	1 (1.8%)
Recipient cause of ESRD	
Diabetes Mellitus	26 (48.1%)
Hypertension	11 (20.3%)
Glomerulonephritis	7 (12.9%)
Polycystic kidney disease	6 (11.1%)
Other	4 (7.4%)
Recipient mean EPTS (±SD)	46.5 (±28.1)
Donor mean age, years (±SD)	40.2 (±14.7)
Donor mean KDPI (±SD)	44 (±22.4)

Of the 54 recipients, 36 (66.7%) were male, and 18 (33.3%) were female. The mean age was 53.2 years (range: 27-76). There were 22 (40.7%) African American recipients, 12 (22.2%) Hispanic recipients, 11 (20.3%) Caucasian recipients, eight (14.8%) Asian recipients, and one (1.8%) recipient of “other” race. The etiology of ESRD was diabetes mellitus in 26 (48.1%) recipients, hypertension in 11 (20.3%) recipients, glomerulonephritis (GN) in seven (12.9%) recipients, polycystic kidney disease (PKD) in six (11.1%) recipients, and other causes in four (7.4%) recipients. The mean recipient EPTS score was 46.5% (±28.1). The mean donor age was 40.2 (±14.7) years, and the mean KDPI score was 44% (±22.4). Table 2 shows post-transplant outcomes.

**Table 2:** Post-transplant outcomes (n = 54) SD: standard deviation; DGF: delayed graft function; BPAR: biopsy-proven acute rejection; SCr: serum creatinine

Characteristics	No. (%)
Time from waitlist to transplant, days (±SD)	216 (±204)
DGF	5 (9.2%)
BPAR	3 (5.5%)
Mean SCr at 1 year, mg/dL (±SD)	1.4 (±0.44)
Graft survival at 1 year	52 (96.2%)
Patient survival at 1 year	53 (98.1%)

The mean time from the waitlist to transplant was 216 days (range: 4-792). DGF occurred in five (9.2%) recipients. In the first year after transplant, 13 patients underwent kidney biopsy for cause. Two patients had borderline acute cellular rejection, and one patient had acute antibody-mediated rejection. This patient had a reduction in maintenance immunosuppression for BK viremia and had donor-specific antibodies at the time of biopsy. Of the 10 patients who had no signs of rejection on biopsy, eight were positive for C4d without any features of microvascular inflammation. At one-year post-transplant, the mean serum creatinine was 1.4 (±0.44) mg/dL, while the graft survival rate was 96.2%. Of the two graft failures that occurred in the first year after transplant, one was due to death with a functioning graft, and the other was due to focal segmental glomerulosclerosis, favored to be donor-related and/or secondary to calcineurin inhibitor toxicity. At one-year post-transplant, recipient survival was 98.1%. The only patient death was secondary to delta-variant COVID-19.

## Discussion

Our study showed that A2/A2B to B DDKT is safe and primarily benefited traditionally disadvantaged blood group B minority patients. Our results echoed the findings of other studies that demonstrated the safety of A2/A2B to B kidney transplantation [8-11]. Nelson et al. reported the initial experience of the MTN after the United Network for Organ Sharing (UNOS) approved a voluntary allocation variance prioritizing the allocation of A2/A2B kidneys to B candidates [8]. They reported that between 1994 and 2000, 34% (41 of 121) of blood group B candidates received A2/A2B kidneys, with similar five-year graft survival rates compared to B to B transplants. A retrospective, single-center, cohort analysis of 29 consecutive A2 to B and 50 B to B DDKTs from December 2014 through December 2017 found comparable outcomes between the two groups, though A2 to B transplants were associated with significantly increased costs [9]. Azzi et al. conducted a single-center study of 41 recipients of A2-incompatible kidney transplants against a control group of 75 blood group B recipients between May 2015 and September 2019 [10]. Utilizing an anti-A2 titer of <1:16, they demonstrated that A2-incompatible transplantation is safe. This study also showed that C4d positivity in graft biopsies is common but did not correlate with acute rejection. Lum et al. reported a study of 49 blood group B candidates who received A2/A2B kidneys at their center and found an 83.6% increase in transplant volumes for blood group B waitlisted patients and a 22.5% decrease in waiting time for transplantation, with similar post-transplant outcomes [11].

The transplantation of deceased donor A2/A2B kidneys to blood group B patients and its influence on wait times was assessed retrospectively in 1,400 kidney transplants by Bryan et al. [12]. With 56% A2/A2B to B transplants in their study, they reported that median wait times were similar among groups (A2/A2B to B = 182 days and B to B = 297 days), with 72% seven-year actuarial graft survival. Similarly, the impact of the 2002 OPTN policy change and variance in practice that allowed the allocation of A2/A2B deceased donor kidneys to blood group B recipients was reported by Williams et al. [13]. Across eight donor service areas, 101 blood group B patients received A2/A2B kidney transplants through 2011, increasing the access of blood group B minority candidates to kidney transplantation, with comparable three-year graft survival rates. Additionally, Scientific Registry of Transplant Recipients (SRTR) data from 2013 to 2017 was used to conduct a case-control study of blood group B recipients of A2/A2B kidneys vs. non-A2/A2B kidneys [14]. The analysis reported a 4.9-fold increase in A2-incompatible DDKT since the introduction of the KAS in 2014, although transplantation rates among minority recipients did not improve compared to Caucasian recipients. Most recently, Bisen et al., using SRTR data from 2014 through 2022, identified 1,897 patients who received A2/A2B to B DDKT and reported similar rates of graft failure and mortality compared to B to B compatible recipients [15].

Despite a notable history of safety and subsequent policy changes to preferentially allocate A2/A2B kidneys to eligible B candidates, only about a third of transplant centers in the U.S. are currently performing A2/A2B to B kidney transplantation [6,15]. Continued reports of its safety and efficacy could raise awareness among transplant centers and encourage increased uptake of A2/A2B to B DDKT in the U.S. However, additional factors are likely contributing to the low uptake. Reporting of A1/A2 subtyping among blood group A and AB deceased donors by Organ Procurement Organizations (OPOs) was only 56% as of 2021 [15]. A study by Garg et al. identified significant misclassification in A2 donor genotyping, revealing that 49.6% of A2 donors were incorrectly subtyped as A1 at donor centers, resulting in a substantial lost opportunity for transplantation [16]. Another study analyzed 554 deceased blood group A donor samples and compared lectin-based with genotype subtyping from two transplant laboratories, and found that genotyping identified 65% more A2 donors than lectin-based subtyping, which is the current standard for blood group A subtyping in transplantation [17]. Another factor that might be contributing to the low uptake of A2/A2B to B DDKT is the lack of consensus on an acceptable anti-A titer threshold among B candidates to be deemed eligible to receive A2/A2B kidneys. Also, there is a lack of consensus on testing approaches, i.e., anti-A titer vs. anti-A2 titer, total antibody, and/or IgM vs. IgG only. Our center has utilized an anti-A IgG titer with a cut-off threshold of <1:8. We chose this threshold based on the data from early work in this field by MTN [2]. A recent study by El Chediak et al. showed no differences in long-term graft survival between patients with low vs. high pre-transplant anti-A titers, defined as 1:8 or less vs. 1:16 or greater [18]. As newer data like these become available, it is imperative for the transplant community to push the envelope by exploring higher titer thresholds to benefit more transplant candidates.

Some limitations of our study include its retrospective nature, with a smaller sample size. Additionally, there was no blood group B donor to blood group B recipient cohort available for comparison of outcomes. Lastly, outcomes at one-year post-transplant were reported, but longer-term post-transplant outcomes were not addressed. Multi-center studies addressing higher anti-A titer thresholds and long-term outcomes are warranted to increase the robustness and generalizability of the findings of our study. 

## Conclusions

Our study showed excellent short-term graft and patient survival rates with A2/A2B to B DDKT. Predominantly, minority patients benefited from this approach. A2/A2B to B kidney transplantation helps lessen racial disparities in kidney transplantation by increasing access for traditionally disadvantaged blood group B minority candidates. Future opportunities include continuing efforts to increase awareness of its safety and improve the reporting of blood group A deceased donor subtyping. Additionally, the development of societal guidelines for anti-A titer testing among blood group B candidates, with the establishment of acceptable eligibility thresholds, might increase the uptake of A2/A2B to B kidney transplantation among transplant centers in the U.S.
